# Current status of haploidentical stem cell transplantation for leukemia

**DOI:** 10.1186/1756-8722-1-27

**Published:** 2008-12-31

**Authors:** Xiao-jun Huang

**Affiliations:** 1 Institute of Hematology, People's Hospital, Peking University, No 11, Xi Zhi Men South Street, Beijing, 10044, PR China

## Abstract

Haploidentical hematopoietic stem cell transplantation has made tremendous progress over the past 20 years and has become a feasible option for leukemia patients without a HLA identical sibling donor. The early complications of severe graft-versus-host disease (GVHD), graft failure and delayed engraftment, as well as disease recurrence have limited the use of this approach. Newer strategies have been applied and overcome some of the problems, including the use of T-cell depleted graft, "mega" dose of stem cells, intensive post-transplant immunosuppression and manipulation of the graft. These have decreased the transplant related mortality and GVHD associated with haploidentical transplantation, however, the major problems of disease relapse and infection, which related to late immune reconstitution, limit the development of haploidentical HSCT. Future challenges remain in improving post-transplant immune reconstitution and finding the best approach to reduce the incidence and severity of GVHD, while preserving graft-versus-leukemia effect to prevent the recurrence of underlying malignancy.

## Background

Hematopoietic stem cell transplantation is a good, and sometimes only treatment option for the cure of leukemia, especially for patients with high risk factors of relapse and with relapsed leukemia[1]. However, only 30% of patients can find an ideal donor, an HLA-identical sibling. The only option is transplantation from an alternative donor. Although the chances of finding an suitable unrelated donor have been significantly increased due to the expansion of the worldwide unrelated donor program, the application of unrelated donor transplantation remains limited by some major obstacles, including 1) the probability of finding a matched unrelated donor(MUD) ranges from less than 10% in ethnic minorities to 60%~70% in Caucasians[2], 2) the complicated process of searching, HLA-typing, and harvesting an unrelated donor takes an average of about 4 months from initiation of a search to the donation of stem cells. Some patients might relapse or even die during this waiting period[3], 3) moreover, allogeneic transplantation using a matched unrelated donor is still associated with a high transplant-related mortality and high long-term morbidity[4,5]. Unrelated donor umbilical cord blood (UCB) offers the advantages of easy procurement and immediate availability, the absence of risk of donor, and potential reduced risk of GVHD[6]. However, engraftment remains a significant problem, especially for adult patients receiving UCB with low number of hematopoietic stem cells and two-antigen mismatch.

The use of mismatched family donors offers several advantages, 1) immediate and easy donor availability, virtually almost all patients have at least one HLA-mismatched relative donor, who is immediately available to serve as a donor; 2) The ability to select the best of many potential donors on the basis of HLA mismatch, age, natural killer (NK) cell alloreactivity[7-9]; 3) Easy access to repeat donation of donor cells again, when donor-derived cellular therapy is needed for the treatment and/or prophylaxis of relapse, or for second transplantation when graft failure or poor engraftment occurred; 4) A potentially stronger graft-versus-leukemia (GVL) effect.

## Haploidentical SCT, an historical perspective and recent advances

Haploidentical/HLA-mismatched stem cell transplantation has been carried out for more than 20 years. Early practice by Fred Hutchinson Cancer Research Center[10] demonstrated the promise and limitation of haploidentical HSCT for leukemia. The overall survival for patients with acute leukemia in remission was not significantly different following HLA-matched and one antigen mismatched donor HSCT, while the outcome of patients received HLA 2 or 3-loci mismatched transplant were poor. Compared to HLA-identical sibling donor transplantation, haploidentical transplantation had a significantly higher incidence of severe GVHD, delayed engraftment and graft failure, which carried a high mortality rate. Their results suggested that transplants involving patients who received 2- or 3-antigen mismatched related donors should be avoided routinely in leukemia, and that haploidentical HSCT using conventional myeloablative conditioning regimen and pharmacological (cyclosporine-based) GVHD prophylaxis was problematic.

Since then, many researchers focused on the techniques of ex vivo T-cell depletion (TCD) of graft. The best survival rates were about 55% for AML and 28%for ALL in adult patients; poor post-transplant immune reconstitution and infection-related mortality remains the major obstacle. Some other centers, such as our center in Beijing, China, focused on manipulating the graft and post-transplant immune suppression. Recently, we reported on 171 patients who underwent transplantation from haploidentical family donors and the disease-free survival (DFS) at 2-year was 68% for standard-risk leukemia and 42% in high-risk patients[11]. Significantly better result was achieved by these protocols than that achieved by TCD. The results of the prominent trials in this regard are shown in Table 1, and are divided according to whether in-vitro TCD was used. The different characters of the two categories are discussed in depth below.

**Table 1:** T cell-depleted and unmanipulated haploidentical stem cell transplantation

Centers (year)	Disease	No. of pts	Conditioning	GVHD prophylaxis	Cell dose 10^6^/kg		TCD methods	Graft failure	Acute GVHD	Chronic GVHD	TRM	Relapse	DFS
					CD34	CD3							

**T-cell depleted**													
Perugia, Italy 1994[16]	AML/ALL/CML	36	TBI/TT/CY/ATG	Extensive TCD	10.8	0.22	Lectin-based+E-rosette	1/17	1/16	0/9	9/17	2/17	6/17

USC, USA 1997[12]	AML/ALL	72	TBI/VP16/Ara-C/CY/ATG	Partial TCD CSP, MP, ATG	1.36	0.075	Ex vivo TCD with T10B9	2%	16%	35%	65.3%	32%	2-year OS 55% for SR 27% for HR

Perugia, Italy 1998[17]	AML ALL	44	TBI/TT/F/ATG	Extensive TCD	10.5	0.02	Ex vivo CD34 selection	5%	0	0	40%	13% 63%	36% 17%

Boston, USA 1999[40]	AML/ALL/NHL/others	12	TBI/Ara-c/CY	CTLA4-Ig, CSP+MTX	2.2	28	CTLA4-Ig,	1/11	3/11	1/11	7/12	1/12	5/12

Multicenter, Japan 2000[61]	Leukemia/others	135	TBI-based	CSP/Tac/MTX/Steroids	3.2 5.5 4.9	0.06(M) 0.09(P) 0.01(M+P)	Ex vivo CD34+ selection	13%	II~IV 21%	35%	47%	22%for SR 79%for HR	5-year 39% for SR 5% for HR

Tuebingen, Germany 2004[22]	AML/ALL/others	63	TBI or Bu-based with F/TT/ATG/Cy	Extensive TCD	19.5	0.011	Ex vivo CD34/CD133 selection	17%	II 7%	13%	28%	30%	3-year 48% (ALL/NHL)

USC, USA 2004[13]	AML/ALL	201	TBI/VP16/Ara-C/Cy/ATG	Partial TCD CSP, MP, ATG	1.9	0.05	Ex vivo TCD with T10B9/OKT3	2%	13%	15%	51%	31%	5-year 18%

Multicenter, Canada, 2004[62]	AML	11	Mel/TT/F/ATG	TCD	13.7	0.005	Ex vivo CD34 selection	0%	0%	NA	55%	37%	9-month 9%

Perugia, Italy 2005[18,21]	AML/ALL/CML	175	TBI/TT/F/ATG	Extensive TCD	12.8	0.01	Ex vivo CD34 selection	6%	II~IV 8%	4%	36.5%	24% for AML 27% for ALL	5-year 47% in CR 4% in rel

Tuebingen, Germany 2007[25]	HM/AA	38	F/TT/Mel/OKT3	TCD	16	0.049	CD3/CD19 negative selection	17%	II~IV 27%			2.6%	70% for SR 20% for HR

Multicenter, Germany 2008[24]	HM	29	F/TT/Mel/OKT3	TCD	7.6	0.044	CD3/CD19 negative selection	0%	II~IV 48%	3/29	8/29	41%	1-year 35%

**Non-T-cell depleted**													

Jone-Hopkins University 2004[37]	Leukemia/MDS	56	F/CY/TBI(2 Gy)	Tac/MMF/CY	NA	NA	Not done	17%	66%	NR	9%(d100)	NA	1-year 38%

Beijing, China 2006[11]	ALL/AML/CML/MDS	171	Bu/Ara-C/CY/Me-CCNU/ATG	CSP/MMF/MTX short	1.8	220	Not done	0	55%	47%	2-year 19% for SR 31% for HR	12% for SR 39% for HR	2-year 68% for SR 42% for HR

Japan, 2006[39]	Leukemia/lymphoma	26	F/Bu/ATG	Tac/MP	6.55	254	In vitro TCD with ATG	4%	19%	25%	15% (d100)		3-year 61%

Duke University, USA 2007[38]	Leukemia/MDS/MPD	49	F/CY/Alemtuzumab	MMF ± CSP Partial TCD	13.5	460	In vivo ± ex vivo TCD/Alemtuzumab	14%	16%	14%	31%	49%	1-year 43%

*AML = acute myeloid leukemia; ALL = acute lymphoid leukemia; Ara-C = cytarabine; ATG = anti-thymocyte globulin; BU = busulfan; CML = chronic myeloid leukmemia; CR = complete remission; CSP = cyclosporine A; CY = cyclophosphamide; DFS = disease free survival; F = fludarabine; GVHD = graft versus host disease; HM = hematologic malignancies; HR = high risk; MDS = myelodysplastic syndrome; Mel = melphalan; MMF = mycophenolate mofetil; MP = methylprednisolone; MTX = methotrexate; NA = not available; NHL = non-Hodgkin lymphoma; OS = overall survival; pts = patients; rel = relapse; SR = standard risk; Tac = tacrolimus; TBI = total body irradiation; TCD = T-cell depletion; TRM = transplant related motality; TT = thiotepa.

## T cell depleted graft

### Partial T-cell depletion

Hanslee-Downey and colleagues[12] explored a novel sequential immunomodulation in haploidentical transplantation, combining ex vivo TCD with the T10B9 monoclonal antibody (mAb) and in vivo T cell lysis with immunotoxin H65-RTA, which was replaced by antithymocyte globulin (ATG) in their large study. Seventy two patients received haploidentical transplant following TBI-based conditioning. Post-transplant GVHD prophylaxis comprised cyclosporine (CSP), steroids and ATG. The 88% engraftment rate, 16% incidence of grade II~IV acute GVHD and 35% incidence of chronic GVHD were acceptable, and Two-year DFS for high-risk and low-risk patients were 23% and 53%, respectively. In their later report[13], 201 patients with acute leukemia received grafts from haploidentical donors with partial in vivo T-cell depletion by T10B9 mAb (n = 143, 1993–1994) or by OKT3 (n = 58, 1995–1999).

The median number of T cell in graft was 5 × 10^4^/kg, which was 1 to 1.5 log lower than unmanipulated graft. Engraftment was successful established in 98% of patients. 13% incidence of grade II~IV acute GVHD and 15% incidence of chronic GVHD were encouraging. Unfortunately, the 5-year overall survival (OS) was only 19%, and 5-year cumulative incidences of relapse and transplant-related mortality (TRM) were 31% and 51%, respectively. Their series studies highlighted the efficacy of partial TCD in preventing GVHD in mismatched transplantation and the potential of post-grafting immunomodulation in lowering the risk of graft failure.

### Extensive TCD with "Megadose" stem cells

Given the key role of the cell dose in graft which was associated with transplant-related mortality and disease-free survival[4,14], Another attempt to overcome the major obstacles of mismatched HSCT in leukemia was to increase stem cell numbers in graft. With the pioneering work of Reisner[15], Aversa and his group produced a series of clinical trials, administered "megadose" of donor hematopoietic stem cells by combining G-CSF mobilized peripheral blood with bone marrow stem cells, both depleted of T cells, following TBI-based conditioning regimens[16-18]. In their early report, the median numbers of CD34+ and CD3+ cells were 10.8 × 10^6^/kg and 0.22 × 10^6^/kg, respectively. Although engraftment was prompt with low occurrence of GVHD, subsequent follow-up revealed problems of both late rejection and/or graft failure, which led to high TRM[16].

The availability of techniques for positive selection of CD34 cells by using the CliniMACS system has provided enriched CD34+ stem cells with a powerful and reproducible TCD (median 4.5 logs) in grafts. The median cell numbers in Aversa's report were 12.8 × 10^6^/kg for CD34+ and 0.01 × 10^6^/kg for CD3+ cells, respectively[18]. CD34 positive selection also provided a median 3.2-log B-cell depletion, which helped prevent EBV-related lymphoproliferative disorders in patients who received an extensive T-cell depleted HSCT after a conditioning regimen with ATG[19,20]. They got a favorable outcome in this large series of high-risk acute leukemia (n = 175, data updated in 2008[21]), with a 95% engraftment rate and 8% incidence of acute GVHD. However, DFS was only 25% for ALL in CR and better for AML patients (40% and 50% for ≥ CR2 and CR1, respectively) which related to disease status at transplantation. High non-relapse mortality (NRM) of about 40%, the majority of which was due to infection, remained the usual obstacle harming post-transplant survival.

CD133 is mainly co-expressed with CD34+ and also found in CD34- precursors, which have higher clonogenic potential. Lang et al, from Tuebingen, Germany, reported their data during a 9-year period of 63 pediatric patients receiving CD34+ or CD133+ selected stem cells from mismatched family donors, following a TBI- or Busulfan-based conditioning regimen[22]. Patients received a median of 19.5 × 10^6^/kg purified cells and 0.011 × 10^6^/kg CD3+ T lymphocytes, without regular post-transplant pharmacological immunosuppressive agent. 83% achieved stable primary engraftment. Grade II acute GVHD (aGVHD) occurred in only 7% of patients and no severe aGVHD were seen. The 48% of long-term survival for patients with ALL in remission was promising. However, the outcome was poor for AML/CML (3-year DFS 18%).

Although "Megadose" stem cells have been proved to facilitate stable engraftment despite such an extensive T-cell-depleted stem cell graft by several groups for haploidentical HSCT[21,22], the rate of rejection increased and kinetics of engraftment and immune reconstitution were delayed at doses below 10 × 10^6^/kg, particularly when less than 8 × 10^6^/kg CD34 cells[23]. To harvest higher number of CD34 cells may place considerable demand on not only donors but also the cell pheresis service, especially for adult patients with high body weight. This kind of approach is still complicated by a high TRM due to the delayed immune reconstitution and the intensive conditioning regimen used. In fact, the difficulty in attaining rapid immune reconstitution is still an unresolved problem for haploidentical transplant.

### Negative selection of CD34 cells

The Tuebingen's group developed a novel strategy aiming at improving engraftment in haploidentical transplant, even with lower numbers of CD34+ cells. CD3/CD19 depleted grafts were made by CD3- and CD19-coated microbeads on a CliniMACS system[24,25]. T- and B-cell depletion was profound up to 4.4 logs. Reduced intensity conditioning was used, including fludarabine (150~200 mg/m^2^), thiotepa (10 mg/kg), melphalan (120 mg/m^2^), and OKT-3 (5 mg/day, -5 to +14). Rapid engraftment was seen at a median of 12 days (range 10–21) for granulocytes and 11 days (range 7–38) for platelets, and full donor chimerism after 2 to 4 weeks in all 29 patients. Although patients received a much lower dose of CD34+ cells (median 7.6 × 10^6^/kg) compared to CD34 positive selection, the grafts contain high numbers of CD34 negative cells, such as NK cells, monocytes and antigen-presenting cells that have engraftment facilitating properties. There is, however, a higher incidence and degree of GVHD, 48% of grade II~IV acute GVHD, after CD3/CD19 depleted transplant than that of CD34 positive selection (lower than 10%). This may due to the difference in CD3+ cell dose in grafts by using the CD34 positive or negative selection techniques. TRM was 28% and OS was 34%, with only 9 patients in completed remission with a follow-up of 241 days.

### Selective TCD

As shown above, extensive T-cell depletion successfully prevented severe acute GVHD, but resulted in immunodeficiency which related to disease recurrence and an increased risk of infections. Simple T cell infusion may not be a good idea to overcome these barriers, one of the most promising approaches is to selectively remove GVHD-causing alloreactive T cells, while keeping cells mediating the graft versus leukemia (GVL) effect and antimicrobial immune responses in grafts[26]. Some pioneering clinical trials have demonstrated its availability and feasibility[27,28]. Amrolia's group used an anti-CD25 immunotoxin to deplete alloreactive lymphocytes in 16 haploidentical transplantation patients. Higher-dose group showed significantly improved T cell recovery and earlier recovery of CMV- and EBV-specific responses compared to lower dose group, particularly at 3–5 month post transplant[28]. Other studies of allodepletion have been reported to reduce GVHD by using anti-CD95[29], photodepletion[30] in murine models and humans[31]. However, there are several concerns and limitations should be kept in mind. First, selective depletion techniques are complex and expensive in time and materials, which need to be improved and simplified before routine use in clinical transplant centers. Second, despite the low number, the complete elimination of alloreactive T-cell subsets could be difficult, and the best technique and the optimal T cell number remain to be determined. Third, the patients who underwent a selective allodepleted transplant may be at risk of GVHD.

### Other reports of TCD

There are some other reports of TCD that could be found in Table 1.

## Non in vitro TCD transplantation

Recently, we reported on an intensive in vitro immunosuppressive protocol without in vitro TCD for haploidentical transplantations, in Beijing, China[1,11,32]. Patients were conditioned with modified Busulfan plus cyclophosphamide regimen and received combined granulocyte-colony stimulating factor primed bone barrow (G-BM) and peripheral blood stem cells (PBSC) graft from haploidentical family donors. CSP, mycophenolate mofetil (MMF), and methotrexate (MTX) were given as GVHD prophylaxis. All 171 patients, including 86 in high-risk group attained sustained full donor chimerism. Although the T cell dose in graft was more than 100 × 10^6^/kg, the incidence of grade III~IV aGVHD and extensive chronic GVHD were acceptable, 23% and 47% respectively. The 2-year probability of relapse was 12% for standard-risk disease and 39% for high-risk disease. 65% of patients were alive without leukemia recurrence during a median follow-up of 682 days, and 2-year leukemia free survival was satisfactory for the standard-risk and high-risk group (68% vs 42%, p = 0.0009), which was comparable with that of HLA-matched sibling donor transplantation^11^. The good results could be related to several factors. First, large numbers of T cells infused were partially contributed to the efficient engraftment when the number of CD34+cells (1.8 × 10^6^/kg) was not high. Second, the intensive, sequential immunosuppression, including ATG, was related to the high engraftment and low incidence of GVHD, Third, the hyporesponsiveness of T cells and the immunomodulatory effect of T-polarized cells (Th2) from the G-CSF primed bone marrow graft and PBSC, respectively[33-35].

Researchers at Johns Hopkins University have focused their efforts on using post-transplant administration of high-dose cyclophosphamide (50 mg/kg) as a way to decrease the rates of both graft rejection and acute GVHD following non-TCD haploidentical transplantation. In their phase I clinical study, 8 of 13 patients achieved sustained donor engraftment, 6 developed acute GVHD, and 6 were alive at a median follow-up of 191 days, including 5 in completed remission. Of note, anti-tumor responses were also seen following graft rejection in two of their patients with myelodysplastic syndrome[36]. In 2004, Fuchs et al, presented an updated series involving 56 patients with a variety of advanced hematological malignancies treated with fludarabine, cyclophosphamide and TBI as conditioning. Beside of 1 or 2 doses of cyclophosphamide, tacrolimus and MMF were added as GVHD prophylaxis. Graft rejection only occurred in 9 patients, grade II~IV GVHD occurred in 43% of patients receiving 2 doses of cyclophosphamide, and 78% in patients who received 1 dose. At a median follow-up of 172 days, 21 patients (37.5%) were alive and disease free[37].

Rizzieri et al[38], from Duke University, reported their experience of haploidentical transplantation without ex vivo TCD, in which alemtuzumab was used for in vivo T-cell depletion of both host and donor, in order to allow reliable engraftment and decreased GVHD. A large series of adult patients (n = 49) with hematologic malignancies or marrow failure were enrolled. The conditioning regimen consisted of fludarabine and cyclophosphamide, post transplant GVHD prophylaxis included of alemtuzumab with MMF ± CSP. Their group reported successful engraftment in 94% of patients, low TRM rates of 10.2% and severe GVHD of 8%. Encouraging evidence of quantitative lymphocyte recovery through expansion of transplanted T cells was noted by 3 to 6 months. With most patients in advanced disease at transplantation, 75% of patients attained a complete remission. One-year survival rate of 31% was encouraging, with a median 4.5 years follow-up. Of note, in Rizzieri' report, median dosage of CD34 is 13.5 × 10^6^/kg,

A Japanese study included 26 patients who underwent conditioning with fludarabine, busulfan and ATG followed by a non-TCD haploidentical PBSC transplantation. Tacrolimus and corticosteroids (methylprednisolone 1 mg/kg/day) were used post-transplant as GVHD prophylaxis. All patients but one achieved full donor chimerism. Ten patients developed GVHD, only five with grade II. And 3-year disease-free survival was 61%[39].

### Ex vivo Induction of alloantigen-specific tolerance

Based on in vitro studies[40], Guinan et al evaluated an approach of alloantigen-specific tolerance. Donor marrow was harvested and cocultured with irradiated host mononuclear cells in the presence of CTLA-4-Ig. Twelve patients with advanced hematological malignancies received haplotype bone marrow containing a median of 28 × 10^6^/kg of CD3+ T cells that had been treated in this way after conditioning with TBI plus cytarabine needs to be filled in. Full donor chimerism was obtained in 8 of 10 patients in whom median time to engraftment was 20 days. Only 3 patients developed gastrointestinal GVHD, and no deaths were attributed to GVHD. At the time of reporting, 5 of 12 patients were alive and in remission for 4.5 to 29 months.

## How to improve the results of haploidentical transplant

### Donor lymphocyte infusion

Disease recurrence is still a major complication of haploidentical transplant, sometimes over 50% in high-risk patients. Beside of the high proportion of patients in advanced disease in most series, the high relapse rate is associated with delayed immune reconstitution and lack of graft versus leukemia (GVL) effect. The best management of leukemia relapses after haploidentical HSCT is uncertain. Donor lymphocyte infusion (DLI) has been shown to exert a GVL effect and has been successfully used for treatment of leukemia relapse in patients who have undergone HLA-matched, related or unrelated HSCT[41-44]. There is still less experience in the use of unmanipulated DLI in haploidentical transplant[13,45,46]. Recently, we found that G-CF-primed PBSC instead of unprimed lymphocyte exhibited a comparable or even stronger GVL effect and comparable or reduced incidence of GVHD, and rarely results in pancytopenia[41] When combined with the use of short-term immunosuppressive therapy, such as short-term CSA, or methotrexate for GVHD prophylaxis, the incidence of fatal GVHD complicated with DLI decreased further[42], Based on this outcome, the strategy against leukemia recurrence was converted from therapeutic DLI to prophylaxis DLI for those with advanced hematological malignancies. Twenty nine patients with advanced leukemia received prophylactic DLI at a median 75 (33–120) days after mismatched HSCT. Six patients developed grade 3–4 acute GVHD, and 11 were alive without leukemia relapse for median 932 (250–1567) days[47].

Besides our attempt in using G-CSF mobilized DLI and immunosuppressive therapy, other researchers have developed partially T-cell depleted DLI[48], and purified donor NK cell infusion[49,50]to improve the outcome in high-risk patients who underwent haploidentical HSCT. The primary results are promising.

The diverse results of DLI, including of efficacy, adverse events and survival, is more a reflection of the heterogeneity of patients being treated. And due to the high incidence and severity of GVHD, the safest approach of DLI after haploidentical HSCT, including cell dose, time of infusion, whether using immune suppression, is to be determined. Of note, DLI has a better outcome when exerted in early stage of relapse [51].

### NK cell/KIR ligand mismatching

Lysis of leukemia cells by natural killer (NK) cells is mediated in part by mismatching of the killer immunoglobulin-like receptor (KIR) ligand between the NK cell and its target. Many clinical reports have shown the importance of NK cell alloreactivity between donors and recipients in predicting the prognosis of HSCT[8,9,52]. Some data support a beneficial effect of KIR mismatching. Ruggeri et al found lower relapse risks for patients with AML who underwent TCD haploidentical HSCT, 75% versus 0% for KIR ligand-matched versus mismatched transplant[8]. Leung et al[53] reported similar result of reduced relapse rate in ALL patients. We analyzed the HLA and KIR genotype of 64 donor-recipient pairs, who underwent transplantation, and found that the cumulative incidence of DFS, OS and TRM were best predicted by the number of KIR ligands carried by patients. The KIR ligand-ligand mismatch model was a good predictor of acute GVHD[9]. However, several others suggested a negative effect on clinical outcome[54,55]. The conflicting results might be related with the different protocols of transplant centers and the different definition of NK alloreactivity. Furthermore, the dose of T cell in allograft could be another reason. Cooley et al's report demonstrates that large number of T cells inhibit NK cell function and KIR expression after unrelated HSCT[56]. These data point out the need of further study under different transplant procedures and conditions.

### Regulatory T cells enhancing immune reconstitution

How to improve immune reconstitution remains a substantial challenge in haploidentical transplant. Recently, CD4+CD25+ regulatory T cells were shown to play a major role in tolerance induction to allogeneic responses and used as cellular therapy in order to enhance engraftment and reduce GVHD while preserving GVL effects. Preliminary experience using expanded Tregs in humans is promising, although clinically meaningful expansion of this small subset of effector cell and its beneficial use otherwise acute GVHD have yet to be determined[57,58]. New approaches of cell therapy, including NK/Tregs[59] and MSC[60] infusion, have recently been used in human clinical trial. Although the exact mechanism of immunomodulatory effect of these cells is not clear, encouraging results suggest that acute GVHD may be reduced while retaining GVL effect.

## How to choose from alternative donors?

For patients requiring an allograft but without a HLA-identical sibling donor, the best choice of alternative stem cells remains difficult and controversial. Because of lacking randomized comparisons, when selecting the best alternative donor and type of regimen, many aspects should be considered, including age, disease status, performance status, HLA typing, financial status, urgency of the transplant and availability of donor. Of cause, the experience of transplant center is also an important aspect. Matched-unrelated donor (MUD) has been accepted worldwide and increased rapidly during the past two decades. If there is a readily available unrelated donor matched at 10 out of 10 of A, B, C, DRB1 and DQ alleles, this option should be the first choice. However, high TRM and severe GVHD which lead to morbidity and mortality remains the problem. The long time interval required to identify and acquire donor stem cells is another disadvantage, especially for patient who is in urgent of transplantation. Unrelated cord blood has the advantage of easy procedure and immediate availability. Current data show that a cord blood with a sufficient cell number and more than 4 out of 6 of A, B and DRB1 loci is also a good alternative source for stem cells. However, the low cell dose of cord blood results in poor engraftment in adult patients with a high body weight, though the use of double unit cord blood transplantation and, possibly, ex vivo cord blood expansion, could overcome this difficulty in some patients. Haploidentical transplantation is a relatively new style offering high cell dose, almost unlimited donor availability without time-restriction for transplant, and the potential for graft engineering.

Based on the previous studies, if an allo-graft is crucial for disease treatment, partially matched family donor could be an option for patients without perfectly matched unrelated donors. Sometimes, haploidentical HSCT is even a better choice in experienced transplant center and under the specialized circumstances: 1) urgency for early transplant, such as acute leukemia, but without available HLA-matched donor, 2) post-transplant DLI is highly recommended due to high-risk of leukemia relapse, 3) ethnic minority, in whom the chances of finding an available matched unrelated donor are very low, 4) Patients with a relatively large body weight for whom no cord blood unit with a suitable match and cell dose could be found.

## What is the future direction?

Haploidentical HSCT provides an opportunity for patients to benefit from HSCT when a HLA matched donor is not available. The final goal of haploidentical transplant is to successfully overcome the HLA barrier and capture an optimal GVL effect without GVHD. There are several novel approaches which may be promising in the future 1) selective but effective allodepletion which facilitate successful donor engraftment, improvement of post-transplant immune reconstitution while reducing the incidence of GVHD; 2) improvement of DLI, in order to acquire GVL effect without or limiting GVHD; 3)adoptive cellular immunotherapy, such as Tregs, NK/Tregs, MSCs and donor-derived NK, as well as the third-party cells infusion; 4) pathogen- or leukemia-specific donor-derived T cell infusion could be an additional approach to prevent opportunistic infection and reduce leukemia relapse rate after haploidentical transplant.

## Competing interests

The author declares that they have no competing interests.
